# A Smartphone-Based Information Communication Technology Solution for Primary Modifiable Risk Factors for Noncommunicable Diseases: Pilot and Feasibility Study in Norway

**DOI:** 10.2196/33636

**Published:** 2022-02-25

**Authors:** Inger Torhild Gram, Guri Skeie, Sunday Oluwafemi Oyeyemi, Kristin Benjaminsen Borch, Laila Arnesdatter Hopstock, Maja-Lisa Løchen

**Affiliations:** 1 Norwegian Centre for E-health Research University Hospital of North Norway Tromsø Norway; 2 Department of Community Medicine Faculty of Health Sciences UiT The Arctic University of Norway Tromsø Norway

**Keywords:** eHealth, feasibility study, modifiable risk factor, noncommunicable disease, pilot study, smartphone-based information communication technology solution, short text message service, feasibility, risk, factor, information communication technology, smartphone, development, monitoring

## Abstract

**Background:**

Cardiovascular diseases, cancers, chronic respiratory diseases, and diabetes are the 4 main noncommunicable diseases. These noncommunicable diseases share 4 modifiable risk factors (tobacco use, harmful use of alcohol, physical inactivity, and unhealthy diet). Short smartphone surveys have the potential to identify modifiable risk factors for individuals to monitor trends.

**Objective:**

We aimed to pilot a smartphone-based information communication technology solution to collect nationally representative data, annually, on 4 modifiable risk factors.

**Methods:**

We developed an information communication technology solution with functionalities for capturing sensitive data from smartphones, receiving, and handling data in accordance with general data protection regulations. The main survey comprised 26 questions: 8 on socioeconomic factors, 17 on the 4 risk factors, and 1 about current or previous noncommunicable diseases. For answers to the continuous questions, a keyboard was displayed for entering numbers; there were preset upper and lower limits for acceptable response values. For categorical questions, pull-down menus with response options were displayed. The second survey comprised 9 yes-or-no questions. For both surveys, we used SMS text messaging. For the main survey, we invited 11,000 individuals, aged 16 to 69 years, selected randomly from the Norwegian National Population Registry (1000 from each of the 11 counties). For the second survey, we invited a random sample of 100 individuals from each county who had not responded to the main survey. All data, except county of residence, were self-reported. We calculated the distribution for socioeconomic background, tobacco use, diet, physical activity, and health condition factors overall and by sex.

**Results:**

The response rate was 21.9% (2303/11,000; women: 1397/2263; 61.7%, men: 866/2263, 38.3%; missing: 40/2303, 1.7%). The median age for men was 52 years (IQR 40-61); the median age for women was 48 years (IQR 35-58). The main reported reason for nonparticipation in the main survey was that the sender of the initial SMS was unknown.

**Conclusions:**

We successfully developed and piloted a smartphone-based information communication technology solution for collecting data on the 4 modifiable risk factors for the 4 main noncommunicable diseases. Approximately 1 in 5 invitees responded; thus, these data may not be nationally representative. The smartphone-based information communication technology solution should be further developed with the long-term goal to reduce premature mortality from the 4 main noncommunicable diseases.

## Introduction

Cardiovascular diseases, cancers, chronic respiratory diseases, and diabetes are commonly grouped as the main noncommunicable diseases as they are the world’s biggest killers [1-3]. These noncommunicable diseases share 4 modifiable risk factors (tobacco use, harmful use of alcohol, physical inactivity, and unhealthy diet). An important part of the United Nation’s Sustainable Development Goal target 3.4 is to reduce premature mortality from the 4 main noncommunicable diseases by one-third relative to 2015 levels, by 2030 [4]. Encouraging reduced tobacco use, less harmful use of alcohol, increased physical activity, and healthy diet are simple and cost-effective measures to reduce premature death and disability from the 4 main noncommunicable diseases [5]. Surveillance of the 4 modifiable risk factors is crucial to be able to prevent and control premature death from the 4 main noncommunicable diseases according to the 2030 Sustainable Development Goal agenda [3].

In 2013, the World Health Assembly, the decision-making body of the World Health Organization, adopted a Global Monitoring Framework for noncommunicable diseases with 25 key indicators to track progress in prevention and control of noncommunicable diseases [6]. Before this, the World Health Organization had already introduced the STEPwise approach [7] for the surveillance of noncommunicable disease risk factors. Step 1 included self-reported demographic and behavioral risk factors as well as history of noncommunicable diseases and related conditions; step 2 included physical measurements; step 3 consisted of biochemical measurements.

Statistics Norway performs annual surveys, with representative samples, on tobacco use [8]. Furthermore, there have been several large population surveys [9-12] conducted in various regions and counties during the last fifty years, repeated at approximately 8-year intervals, which have collected some of the data included in the 3 steps of the STEPwise approach [11,12]. In addition, special surveys have been conducted, usually with 10-year intervals, to collect data on detailed dietary intake [9]. Special surveys on physical and sedentary activity have also been conducted with more than 5-year intervals [10]. In summary, there has been a lack of annual data on tobacco use, the harmful use of alcohol, physical inactivity, and unhealthy diet from a nationally representative sample. The Norwegian legislation on public health work [13] requires counties and municipalities to have an overview regarding risk factors, health conditions, and measures to promote health in their respective populations.

In Norway, more than 95% of individuals aged 16 to 54 years, and between 74% to 88% of those aged 55 to 74 years, have smartphones [14]. Short smartphone surveys have the potential to identify modifiable risk factors for individuals and monitor trends. Our main objective was to develop a smartphone-based information communication technology solution with functionalities for collecting data annually on the 4 modifiable risk factors. The secondary objective was to collect nationally representative data.

## Methods

### Study Design

This pilot study, which included a smartphone-based solution, a website, and 2 smartphone surveys, was developed over a 2-year period and conducted during fall 2019.

### Development of the Smartphone-Based Information Communication Technology Solution

**Figure 1:**
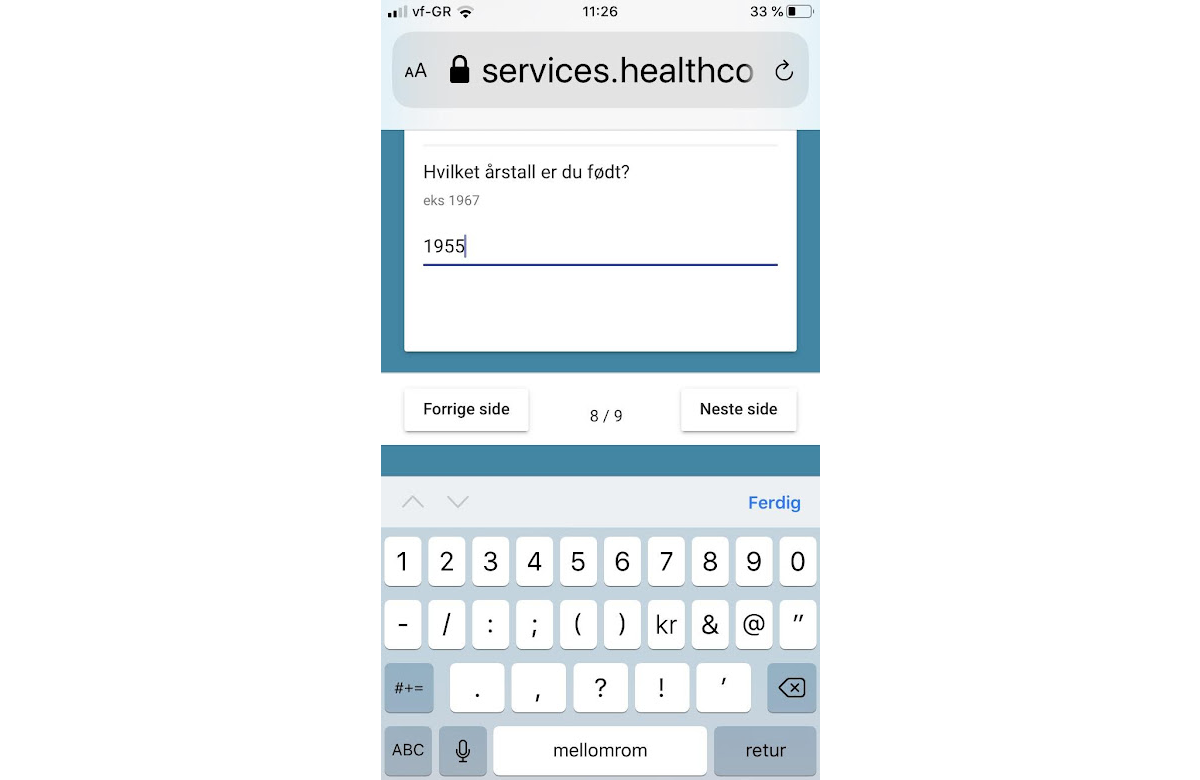
Screenshot of data entry keyboard.

**Figure 2:**
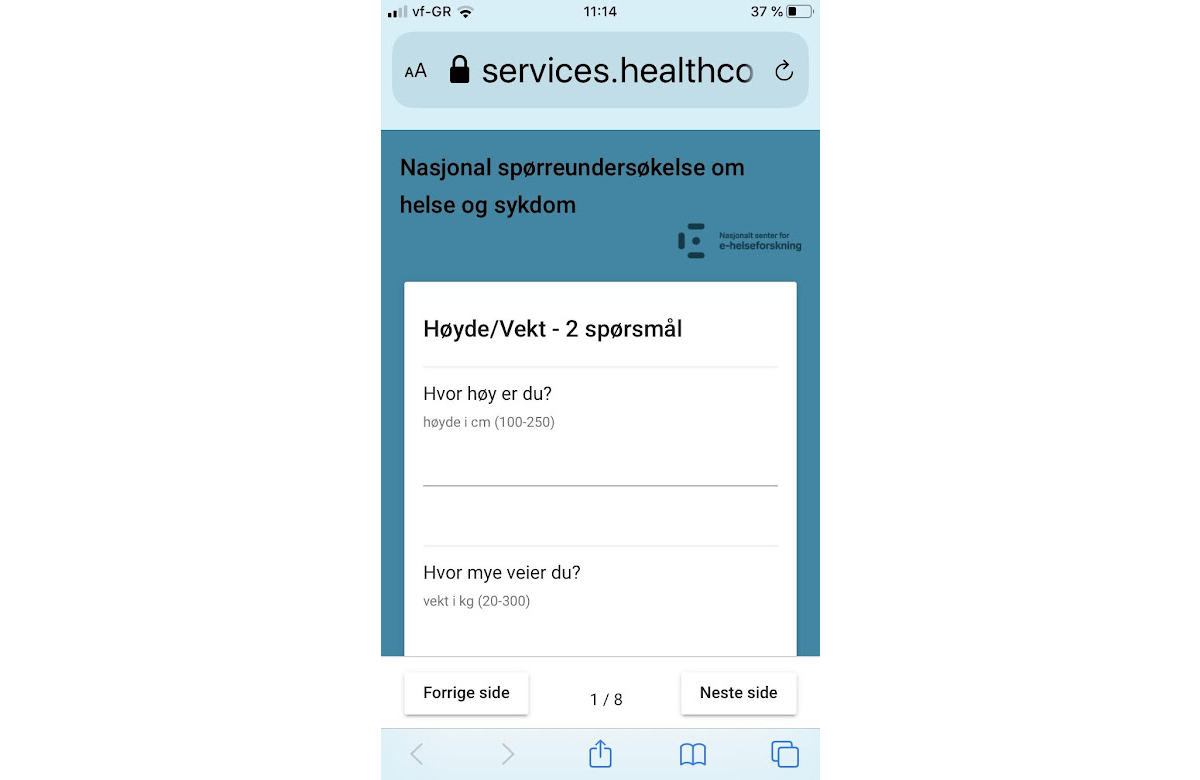
Screenshot of smartphone-based survey.

**Figure 3:**
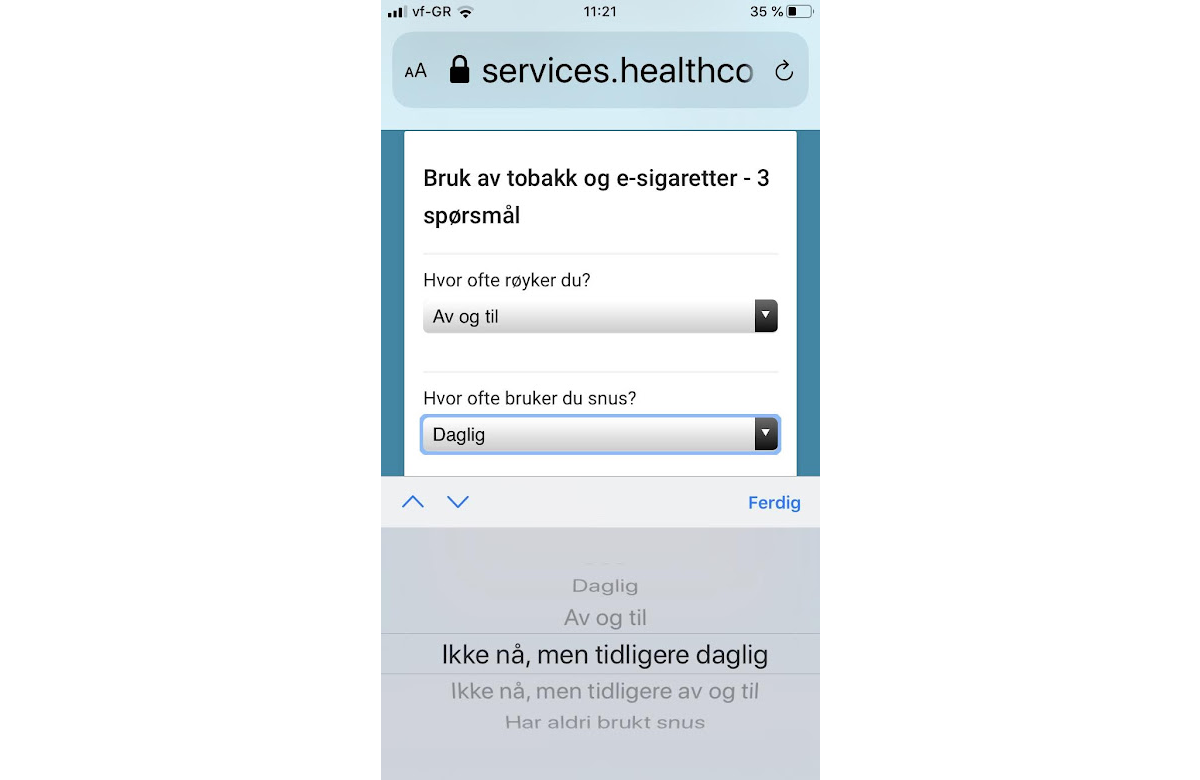
Screenshot of pull-down menus.

The details of the technical and architectural parts of the solution were developed by a private enterprise (Healthcom). For answers to the continuous questions, a keyboard was displayed for entering numbers (Figure 1); there were preset upper and lower limits for acceptable response values (Figure 2). For categorical questions, pull-down menus with response options were displayed (Figure 3).

The information communication technology solution was intended for capturing sensitive data from smartphones and was developed in accordance with general data protection regulations [15]. The cloud-based information communication technology solution automatically created a unique identification number for each respondent, when the initial SMS dialogue started. If the respondent clicked on the survey link later, the respondent’s unique identifier was detected, and the participant could continue to fill in the answers. When the survey was submitted, the responses were anonymized. Subsequently, for analyses and storage, data were transferred to the Research Electronic Data Capture database hosted by the University Hospital of North Norway (Northern Norway Regional Health Authority server system) [16,17]. Communication with the database (over the internet and hospital intranet) was encrypted, and 2-factor authentication was required for researchers retrieving the data.

### Website

**Figure 4:**
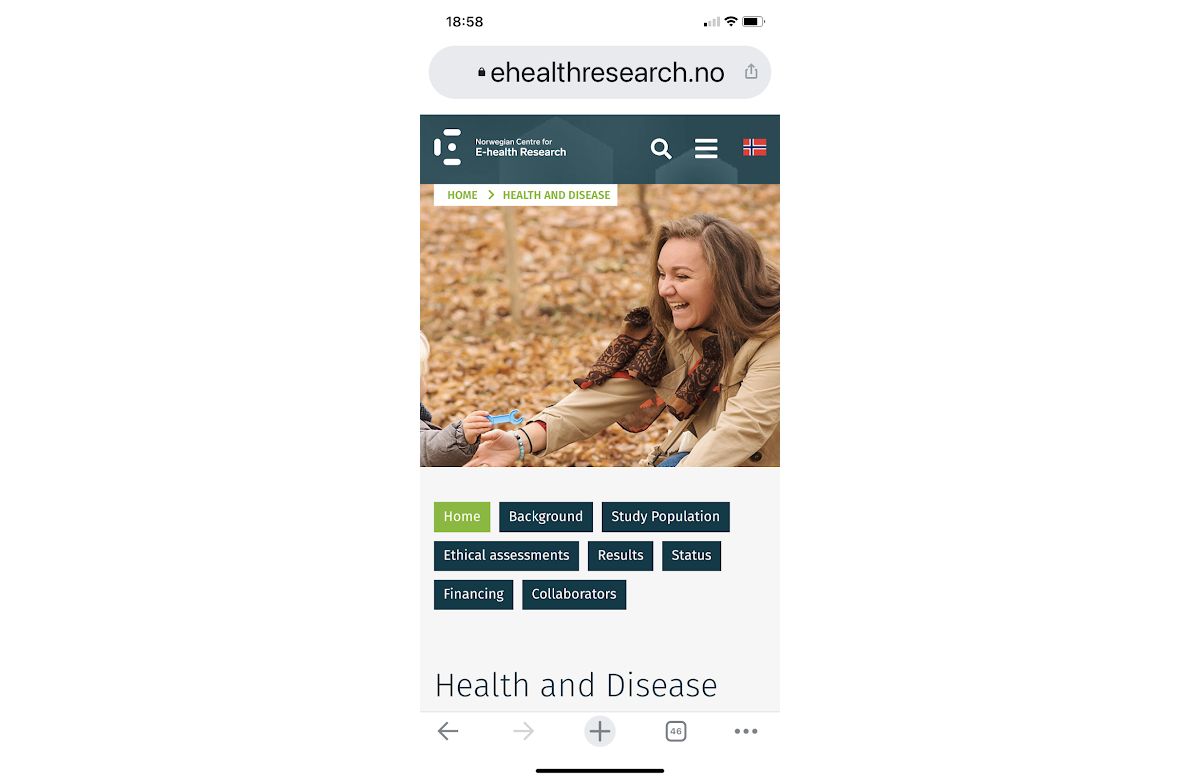
Screenshot of information for invitees.

**Figure 5:**
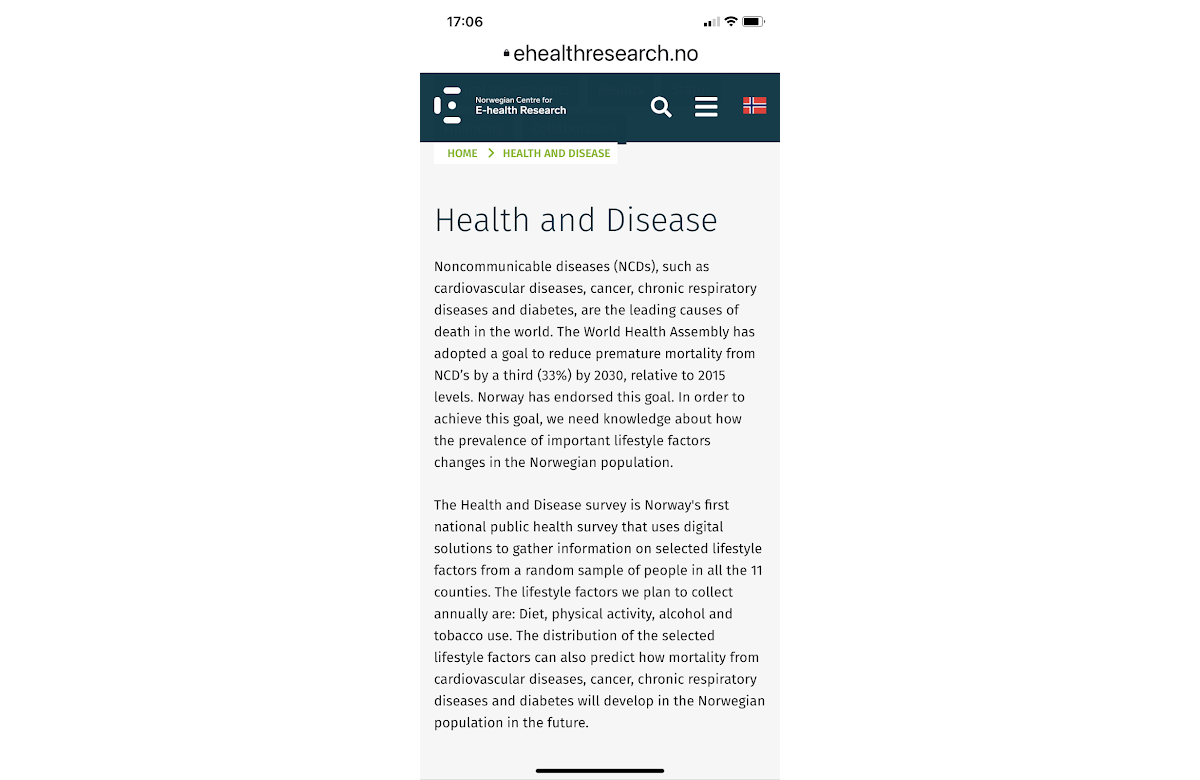
Screenshot of background information.

**Figure 6:**
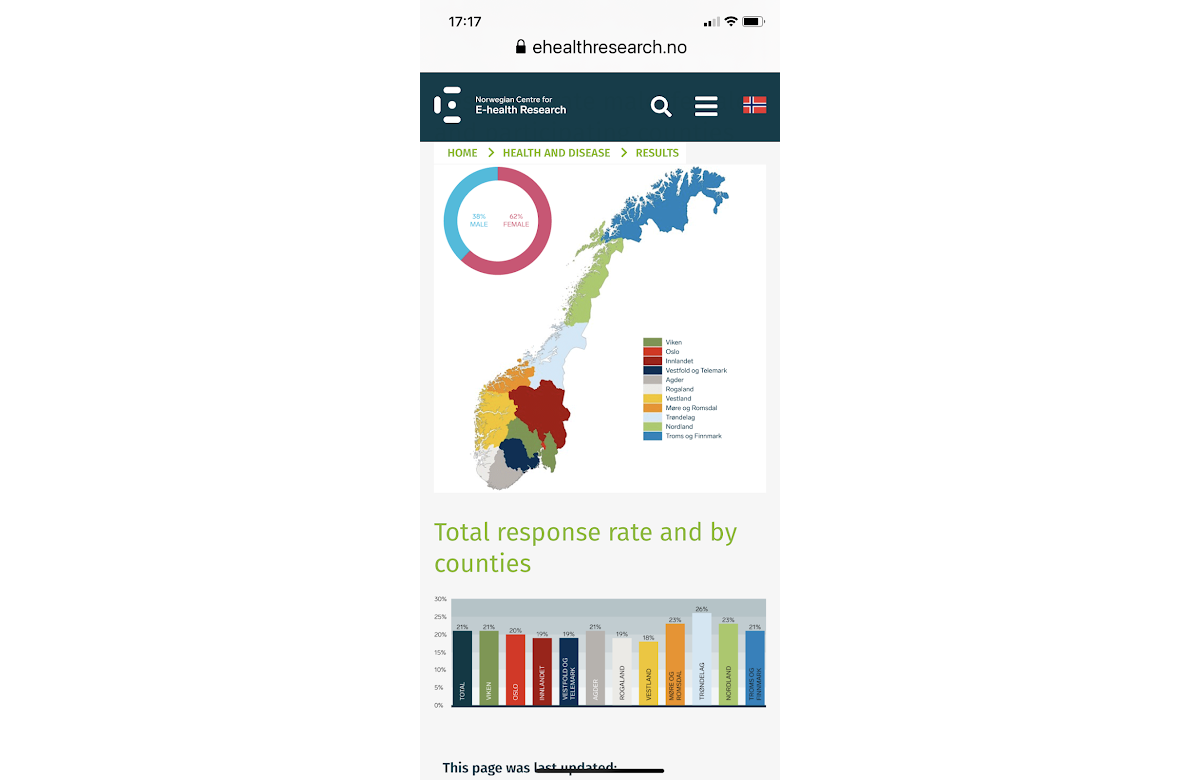
Screenshot of results presented to users.

We developed a website to inform invitees and other interested parties about the study and survey (Figure 4). The website contained information about the background of the study (Figure 5), the study population, ethical assessments, survey results (Figure 6), the status of ongoing plans, study funding, and collaborating partners of the study.

### Smartphone Surveys

#### Overview

We designed 2 mobile phone surveys—the first was sent to all invitees (main), and the second was sent only to a random sample of invitees who had not responded to the main survey. A total of 4 sets of SMS messages were sent: 1 request to participate in the main survey, 2 reminders, and 1 request to participate in the second survey (Multimedia Appendix 1). Invitees could obtain additional information when they accessed the survey and the link to the website (Multimedia Appendix 2).

#### Main Survey

The survey comprised 26 questions in Norwegian. There were 8 questions on socioeconomic background factors: year of birth, sex, education in years (≤10, 11-13,14-16, ≥17), marital status (single, married or cohabitating, divorced, widow or widower), number of persons in the household over and under 18 years (0,1,2,3, ≥4), gross household income in Norwegian Kroner (1 NOK equals approximately US $0.11) during the previous year (≤350,000 NOK, 351,000-550,000 NOK, 551,000-750,000 NOK, 751,000-1,000,000 NOK, ≥1,000,000 NOK), and occupational status (full time work, part-time work, student, retired, home-keeper, military service, and miscellaneous social benefits).

There were 17 questions on the 4 main risk factors. The questionnaire contained 3 questions about cigarette, snus, and e-cigarette use (never, former occasionally, former daily, current occasionally, current daily) and 1 question about alcohol consumption (yes, no). Consumers of alcohol were also asked the first 3 questions from the Alcohol Use Disorders Identification Test [18]—frequency (≤1 time per month, 2-4 times per month, 2-3 times per week, ≥4 times per week), number of units usually consumed (1-2, 3-4, 5-6, 7-9, ≥10), and frequency of occasions of consumption of ≥6 units of alcohol (never, less than monthly, monthly, weekly, daily or almost daily). There were 4 questions related to physical activity from the short version of the International Physical Activity Questionnaire [19]—number of days of strenuous physical activity in the last 7 days (0, 1-2, 3-4, 5-6, 7), number of days of moderate physical activity in the last 7 days (0, 1-2, 3-4, 5-6, 7), number of days of walking for ≥10 minutes in the last 7 days (0, 1-2, 3-4, 5-6, 7), hours spent sitting (excluding sleeping hours) on a regular weekday in the last 7 days (0-2, 3-5, 6-8, 9-11, 12-14)—and 7 questions on dietary intake frequency—servings per day of fruits and berries (0, 1, 2, 3, 4, ≥5), servings per day of lettuce and vegetables (0, 1, 2, 3, 4, ≥5), number of glasses per day sugar-sweetened drinks (0, 1, 2, 3, 4, 5, 6, ≥7), number of times fish and fish products are eaten per week (0, 1, 2, 3, 4, ≥5), number of times red meat is eaten per week (0, 1, 2, 3, 4, ≥5), number of times processed meat is eaten per week (0, 1, 2, 3, 4, ≥5), and how often extra salt is added to food before eating (never, occasionally, often, always).

There was 1 question asking if respondents have or have had any of the following conditions: high blood pressure, high cholesterol, atrial fibrillation, myocardial infarction, heart failure, stroke, chronic respiratory disease, cancer, diabetes, or none of these. In addition, there was an open box for comments. The estimated time for completing the main survey was approximately 5 minutes.

#### Second Survey

The second survey comprised 9 statements that could be answered in the affirmative or negative: “I never answer such surveys”; “I did not want to answer several of the questions”; “I think the questions were difficult to answer”; “I was unsure who the SMS came from”; “I was expecting a login with BankID” (BankID is an electronic identification scheme in Norway for safe log in); “I was afraid that privacy was not taken care of”; “I had not heard of the Norwegian Centre for E-health Research”; “I had not heard of Healthcom”; “I thought the SMS I received looked like spam.” The estimated time for completing this survey was approximately 1 minute. There was also an open box for comments.

The main survey was conducted on 11 consecutive days, with 2 reminders sent 5 and 10 days after the original invitation. The second survey was conducted 1 week after the main survey was completed.

### Ethics

The Regional Committee for Medical and Health Research Ethics concluded that approval for this study was not necessary because the study fell outside the Norwegian Health Research Act. The study was approved by the Data Protection Section of the University Hospital of North Norway. All participants provided consented before answering any survey questions.

### Recruitment

A total of 11,000 individuals (an equal number of men and women) aged 16 to 69 years, with 1000 from each of the 11 counties in Norway, were selected randomly from the Norwegian National Population Registry. All Norwegian residents have an 11-digit personal identification number in the National Population Registry. This registry contains the name, address, sex, age, and mobile phone number of each person. Persons who did not have a registered mobile phone number were replaced. For each person, name, personal identification number, phone number, sex, age, year of birth, and county of residence were retained. Subsequently, the system created a unique survey number for each participant. The Data Protection Section of the University Hospital of North Norway provided a server area specifically for this project where the linkage file could be stored. For the second survey, SMS text messages were sent to a random sample of 100 invitees who had not responded to the main survey from each of the 11 counties. 

### Dissemination

The communication group at the Norwegian Centre for E-health Research sent a standard press release to each county before the launch of the survey. The press release was followed up with phone contact to selected local media in each county. The communication group used social media (Facebook and Twitter) to promote the survey. Several municipalities informed their residents about the survey on municipality website. The project leader also gave interviews on the local radio and to newspapers in some of the counties. In total, the survey was covered in various media channels 127 times.

### Statistical Analysis

All data used in the analyses, except county of residence, consisted of self-reported data. We calculated means (with standard deviations) or medians (with interquartile range) and percentages for each health variable, overall and by sex, using STATA (version 16.0; Stata Corp).

## Results

### Main Survey

Altogether, 25.2% (2769/11,000) participants opened the survey, while 21.0% (2305/11,000) submitted their responses. We excluded 2 participants reporting to be outside the targeted age groups. The remaining 2303 men and women constituted the respondents to the main survey.

The response rate to the main survey was 21.9% (2303/11,000; women: 1397/2303, 60.7%; men: 866/2303, 38.3%; missing: 40/2303, 1.7%). Among men and women, the response rates were 15.7% (866/5500) and 25.4% (1397/5500), respectively. The median age for men was 52 years (IQR 40-61), and the median age for women was 48 years (IQR 35-58). Trøndelag county had the highest response rate (26.0%, 260/1000), and Vestland county had the lowest response rates (18.1%, 181/1000).

Of the 2303 respondents, 1419 (61.6%) answered all the questions; 15 of 29 variables were missing ≤3% values. Each of the questions on socioeconomic background factors had missing values, which ranged from 10.3% (238/2303) for marital status to 20.6% (474/2303) for number of persons under 18 years old in household. Tobacco and alcohol consumption variables had ≤3% missing values, physical activity variables had 1.8% (41/2303) to 9.7% (224/2303) missing values, and food variables had 1.5% (35/2303) to 5.9% (136/2303) missing values (Table 1).

Most respondents were in the oldest (50-69 years) age group (1142/2274, 50.2%), in the highest educational category (graduate or postgraduate university education: 1088/1989, 55.1%), married (1497/2065, 73.1%,), and employed full-time (1158/2015, 57.8%) (Table 2).

Table 3 shows that 55.4% (1225/2209) of respondents reported being either overweight (794/2209, 35.9%) or obese (431/2209, 19.5%), with 63.9% (539/844) of men and 50.3% (686/1365) of women reporting being either overweight or obese.

Daily use of cigarettes (men: 100/855, 11.7%; women: 140/1380, 10.1%), snus (men: 153/850, 18.0%; women: 96/1360, 7.0%), and e-cigarettes (11/848, 1.3%; women: 19/1359, 1.4%) was reported by respondents, and 32.2% (237/735) of men reported drinking alcohol more than once a week, of whom 7.1% (51/724) reported consuming ≥6 units on the same occasion weekly, and 22.7% (254/1119) of women reported drinking alcohol more than once a week, of whom 1.7% (19/1103) reported consuming ≥6 units on the same occasion weekly. Overall, 17.2% (378/2199) reported 0 days with moderate physical activity during the last 7 days (Table 3).

Table 4 shows that 38.5% (875/2268) of respondents reported 1 serving per day of fruit and berries, 48.3% (1091/2255) of respondents reported 1 serving per day of lettuce and vegetables, 67.3% (1458/2167) of respondents reported 0 glasses of sugary drinks per day, 56.6% (1233/2178) of respondents reported ≥2 meals with fish or fish products per week, 67.6% (1465/2167) of respondents reported ≤2 meals of red meat per week, and 23.5% (517/2198) of respondents reported never adding extra salt before eating.

Only 44.5% (984/2209) of respondents were in line with national recommendations for BMI, and 42.0% (932/2219) of respondents were in line with national recommendations for tobacco or e-cigarette use [20]; 81.2% (1501/1848) were in line with national recommendations for alcohol consumption (ie, consuming ≥6 units of alcohol on one occasion less than monthly or never [21]). Only 34.2% (704/2056) of respondents met national recommendations for physical activity levels (ie, walking ≥10 minutes every day the last 7 days [22]), and only 22.9% (522/2281) of respondents met national recommendations for eating fruits and vegetables (ie, at least 5 servings per day [23]).

**Table 1:** Overall respondents and missing values to the main smartphone survey.

Variable	Respondents, N	Missing, n (%)
Age	2274	29 (1.3)
Sex	2263	40 (1.7)
Education	1989	314 (13.6)
Marital status	2065	238 (10.3)
Number of persons (>18 years old) in household	1914	389 (16.9)
Number of persons (<18 years old) in household	1829	474 (20.6)
Gross household income 2018	1946	357 (15.5)
Work life condition/occupation	2015	288 (12.5)
Chronic disease conditions (noncommunicable disease)	2245	58 (2.5)
Height	2270	33 (1.4)
Weight	2247	56 (2.4)
BMI^a^	2237	66 (2.9)
Smoking history	2261	42 (1.8)
Snus use history	2245	58 (2.5)
E-cigarette use history	2234	69 (3.0)
Alcohol consumption (yes/no)	2284	19 (0.8)
Frequency of alcohol consumption^b^	1876	29 (1.5)
Units of alcohol usually consumed^b^	1868	37 (1.9)
Frequency of occasions of consumption of ≥6 units of alcohol^b^	1848	57 (3.0)
Number of days of strenuous physical activity in the last 7 days	2262	41 (1.8)
Number of days of moderate physical activity in the last 7 days	2199	104 (4.5)
Number of days of walking for ≥10 minutes in the last 7 days	2079	224 (9.7)
Hours spent sitting on a regular weekday in the last 7 days (excluding sleeping hours)	2166	137 (6.0)
Fruits and berries intake (servings) per day	2268	35 (1.5)
Lettuce and vegetable intake (servings) per day	2255	48 (2.1)
Sugary drinks, number of glasses per day	2167	136 (5.9)
Fish and fish products, number of times eaten per week	2178	125 (5.4)
Red meat, number of times eaten per week	2167	136 (5.9)
Processed meat, number of times eaten per week	2167	136 (5.9)
Addition of extra salt to food	2198	105 (4.6)

^a^BMI: body mass index.

^b^Among 1905 participants who responded *yes* to alcohol consumption.

**Table 2:** Respondents’ background characteristics (overall and by sex).

Characteristic			All, n (%)^a^		Men, n (%)^a^		Women, n (%)^a^
**Age groups (years)**							
	16-29	347 (15.4)		121 (14.0)		226 (16.3)	
	30-49	760 (33.8)		264 (30.7)		496 (35.7)	
	50-69	1142 (50.8)		476 (55.3)		666 (48.0)	
**Education (number of years)**							
	≤10 (primary school)	219 (11.1)		104 (13.6)		115 (9.5)	
	11-13 (high school)	666 (33.8)		290 (37.8)		376 (31.2)	
	14-16 (graduate)	508 (25.7)		180 (23.5)		328 (27.2)	
	≥17 (postgraduate)	580 (29.4)		193 (25.2)		387 (32.1)	
**Marital status**							
	Single	389 (19.0)		155 (19.7)		234 (18.6)	
	Married or cohabitating	1497 (73.1)		575 (73.1)		922 (73.1)	
	Divorced	126 (6.1)		48 (6.1)		78 (6.2)	
	Widow or widower	36 (1.8)		9 (1.1)		27 (2.1)	
**Number of adults (age ≥18 years) in household**							
	0	151 (7.5)		57 (7.3)		94 (7.7)	
	1	474 (23.6)		184 (23.7)		290 (23.6)	
	2	1013 (50.5)		383 (49.3)		630 (51.3)	
	3	230 (11.5)		93 (12.0)		137 (11.2)	
	≥4	137 (6.8)		60 (7.7)		77 (6.3)	
**Number of children (age <18 years) in household**							
	0	1180 (61.6)		482 (65.1)		698 (59.4)	
	1	293 (15.3)		95 (12.8)		198 (16.8)	
	2	305 (15.9)		115 (15.5)		190 (16.2)	
	3	106 (5.5)		40 (5.4)		66 (5.6)	
	≥4	32 (1.7)		9 (1.2)		23 (2.0)	
**Gross household income in 2018 (in Norwegian Kroner)**							
	≤350,000	249 (12.9)		80 (10.7)		169 (14.3)	
	351,000-550,000	342 (17.7)		117 (15.6)		225 (19.0)	
	551,000-750,000	316 (16.4)		118 (15.8)		198 (16.7)	
	751,000-1,000,000	430 (22.2)		177 (23.7)		253 (21.4)	
	>1,000,000	595 (30.8)		256 (34.2)		339 (28.6)	
**Occupational status**							
	Full-time work	1158 (57.9)		524 (68.1)		634 (51.5)	
	Part-time work	234 (11.7)		37 (4.8)		197 (16.0)	
	Student	146 (7.3)		50 (6.5)		96 (7.8)	
	Retired	180 (9.0)		76 (9.9)		104 (8.5)	
	Other^b^	281 (14.1)		82 (10.7)		199 (16.2)	

^a^Missing responses were not included in calculated percentages.

^b^Home-keeper, military service, and miscellaneous benefits (sick leave, unemployment, disabilities, social security).

**Table 3:** Respondents’ lifestyle factors (overall and by sex).

Factor						All, n (%)^a^			Men, n (%)^a^			Women, n (%)^a^
**BMI^b^ (kg/m^2^)**												
	<18.5				29 (1.3)			3 (0.4)			26 (1.9)	
	18.5-24.9				955 (43.2)			302 (35.8)			653 (47.8)	
	25.0-29.9				794 (35.9)			367 (43.5)			427 (31.3)	
	≥30.0				431 (19.5)			172 (20.4)			259 (19.0)	
**Smoking history**												
	Never				1087 (48.6)			392 (45.9)			695 (50.4)	
	Former occasionally				344 (15.4)			125 (14.6)			219 (15.9)	
	Former daily				469 (21.0)			201 (23.5)			268 (19.4)	
	Current occasionally				95 (4.3)			37 (4.3)			58 (4.2)	
	Current daily				240 (10.7)			100 (11.7)			140 (10.1)	
**Snus use history**												
	Never				1726 (77.8)			571 (67.2)			1155 (84.4)	
	Former occasionally				97 (4.4)			51 (6.0)			46 (3.4)	
	Former daily				80 (3.6)			51 (6.0)			29 (2.1)	
	Current occasionally				66 (3.0)			24 (2.8)			42 (3.1)	
	Current daily				249 (11.2)			153 (18.0)			96 (7.0)	
**E-cigarette use history**												
	Never				2069 (93.7)			786 (92.7)			1283 (94.4)	
	Former occasionally				66 (3.0)			27 (3.2)			39 (2.9)	
	Former daily				15 (0.7)			10 (1.2)			5 (0.4)	
	Current occasionally				27 (1.2)			14 (1.7)			13 (1.0)	
	Current daily				30 (1.4)			11 (1.3)			19 (1.4)	
**Alcohol consumption**												
	No				376 (16.7)			120 (13.9)			256 (18.4)	
	**Yes**				1880 (83.3)			743 (86.1)			1137 (81.6)	
		**Frequency**										
			≤1 time per month	493 (26.6)			149 (20.3)			344 (30.7)		
			2-4 times per month	870 (46.9)			349 (47.5)			521 (46.6)		
			2-3 times per week	402 (21.7)			191 (26.0)			211 (18.9)		
			≥4 times per week	89 (4.8)			46 (6.3)			43 (3.8)		
		**Units usually consumed**										
			1-2	994 (53.8)			366 (49.7)			628 (56.6)		
			3-4	538 (29.1)			215 (29.2)			323 (29.1)		
			5-6	208 (11.3)			86 (11.7)			122 (11.0)		
			7-9	84 (4.6)			56 (7.6)			28 (2.5)		
			≥10	22 (1.2)			14 (1.9)			8 (0.7)		
		**Frequency of occasions with ≥6 units consumed**										
			Never	545 (29.5)			132 (18.2)			403 (36.5)		
			Less than monthly	956 (51.7)			385 (53.2)			561 (50.9)		
			Monthly	277 (15.0)			156 (21.6)			120 (10.9)		
			Weekly	68 (3.7)			49 (6.8)			19 (1.7)		
			Daily or almost daily	2 (0.1)			2 (0.3)			(0.0)		
**Days with strenuous physical activity in the last 7 days**												
	0				639 (28.6)			198 (23.2)			441 (32.0)	
	1-2				828 (37.1)			308 (36.0)			520 (37.7)	
	3-4				527 (23.6)			228 (26.7)			299 (21.7)	
	5-6				180 (8.0)			9 (10.5)			90 (6.5)	
	7				61 (2.7)			31 (3.6)			30 (2.2)	
**Days with moderate physical activity in the last 7 days**												
	0				378 (17.4)			133 (16.0)			245 (18.2)	
	1-2				927 (42.6)			350 (42.1)			577 (42.9)	
	3-4				510 (23.5)			187 (22.5)			323 (24.0)	
	5-6				227 (10.4)			108 (13.0)			119 (8.8)	
	7				133 (6.1)			53 (6.4)			80 (6.0)	
**Days walking for ≥10 minutes in the last 7 days**												
	0				99 (4.8)			55 (7.1)			44 (3.4)	
	1-2				373 (18.1)			154 (19.7)			219 (17.2)	
	3-4				480 (23.4)			189 (24.2)			291 (22.8)	
	5-6				400 (19.5)			153 (19.6)			247 (19.4)	
	7				704 (34.2)			229 (29.4)			475 (37.2)	
**Hours spent sitting on a regular weekday in the last 7 days (excluding sleeping hours)**												
	0-2				162 (7.6)			58 (7.1)			104 (7.9)	
	3-5				876 (40.9)			292 (35.5)			584 (44.3)	
	6-8				598 (27.9)			245 (29.8)			353 (26.7)	
	9-11				348 (16.3)			150 (18.2)			198 (15.0)	
	12-14				109 (5.1)			59 (7.2)			50 (3.8)	
	≥15				48 (2.2)			18 (2.2)			30 (2.3)	

^a^Missing responses were not included in calculated percentages.

^b^BMI: body mass index.

**Table 4:** Respondents’ dietary intake variables (overall and by sex).

Variable		All, n (%)^a^	Men, n (%)^a^	Women, n (%)^a^
**Fruits and berries (servings per day)**				
	0	260 (11.6)	136 (15.8)	124 (9.0)
	1	875 (39.1)	378 (44.1)	497 (35.9)
	2	621 (27.7)	205 (23.9)	416 (30.1)
	3	309 (13.8)	93 (10.8)	216 (15.6)
	4	99 (4.4)	25 (2.9)	74 (5.4)
	≥5	77 (3.4)	21 (2.5)	56 (4.0)
**Lettuce and vegetables (servings per day)**				
	0	134 (6.0)	82 (9.6)	52 (3.8)
	1	1091 (49.0)	494 (58.1)	597 (43.3)
	2	643 (28.9)	190 (22.4)	453 (32.9)
	3	225 (10.1)	50 (5.9)	175 (12.7)
	4	72 (3.2)	19 (2.2)	53 (3.8)
	≥5	63 (2.8)	15 (1.8)	48 (3.5)
**Sugary drinks (glasses per day)**				
	0	1458 (68.1)	497 (60.7)	961 (72.7)
	1	415 (19.4)	192 (23.4)	223 (16.9)
	2	134 (6.3)	67 (8.2)	67 (5.1)
	3	60 (2.8)	28 (3.4)	32 (2.4)
	4	26 (1.2)	10 (1.2)	16 (1.2)
	≥5	47 (2.2)	25 (3.1)	22 (1.7)
**Fish and fish products (number of times eaten per week)**				
	0	151 (7.0)	48 (5.8)	103 (7.7)
	1	769 (35.7)	293 (35.6)	476 (35.8)
	2	789 (36.7)	312 (38.0)	477 (35.8)
	3	339 (15.7)	136 (16.6)	203 (15.3)
	4	82 (3.8)	28 (3.4)	54 (4.1)
	≥5	23 (1.1)	5 (0.6)	18 (1.3)
**Red meat (number of times eaten per week)**				
	0	172 (8.0)	37 (4.5)	135 (10.3)
	1	664 (31.0)	241 (29.3)	423 (32.1)
	2	629 (29.4)	255 (31.0)	374 (28.4)
	3	369 (17.2)	163 (19.8)	206 (15.6)
	4	179 (8.4)	78 (9.5)	101 (7.7)
	≥5	127 (5.9)	49 (5.9)	78 (5.9)
**Processed meat (number of times eaten per week)**				
	0	341 (15.9)	96 (11.7)	245 (18.6)
	1	868 (40.6)	328 (39.9)	540 (40.9)
	2	581 (27.2)	230 (28.0)	351 (26.6)
	3	266 (12.4)	127 (15.5)	139 (10.5)
	4	51 (2.4)	27 (3.3)	24 (1.8)
	≥5	33 (1.5)	13 (1.6)	20 (1.5)
**Addition of extra salt to food**				
	Never	517 (23.8)	192 (23.2)	325 (24.2)
	Occasionally	1264 (58.2)	465 (56.1)	799 (59.5)
	Often	306 (14.1)	127 (15.3)	179 (13.3)
	Always	84 (3.9)	45 (5.4)	39 (2.9)

^a^Missing responses were not included in calculated percentages.

### Second Survey

Altogether, 18.1% (199/1100) of the invitees to the second survey opened it, but 8.2% (90/1100) did not submit a response, while 9.9% (109/1100) replied to some or all and submitted their answers. The latter group constituted the respondents of the second survey. The most common reason for not participating (in the main survey) was “I had not heard of Healthcom” (yes: 43/109, 39.4%; no: 10/109, 9.2%; missing: 56/109, 49.5%), followed by both “I had not heard of the National Center for E-Health Research” and “I was unsure who the SMS came from” (yes: 37/109, 33.9%; no: 11/109, 10.1%; missing: 61/109, 56.0%), then “I thought the SMS I received looked like spam” (yes: 35/109, 32.1%; no: 10/109, 9.2%; missing: 64/109, 58.7%).

## Discussion

### Principal Results

This feasibility study is, to the best of our knowledge, the first successfully piloted smartphone-based information communication technology solution with technically, ethically, and regulatory functionalities for collecting annual data on the 4 main modifiable risk factors for the 4 main noncommunicable diseases. The primary reason reported for not participating in the initial survey was that the sender of the SMS was unknown. Due to the differences in response rates between men and women and between counties, our secondary objective to collect nationally representative data may not have been achieved. If the respondent sample has a similar distribution with respect to background characteristics, life style factors, and dietary intake variables, they may be nationally representative regardless of response rate.

### Comparison With Past Work

We are not aware of any previous work with the same overall objectives as ours.

We chose to use SMS text messaging as the means of initial contact, instead of internet or email surveys, ordinary or computer-assisted telephone interviews, or interactive voice response because text messages are often perceived as personal forms of communication; they are more likely to be read quickly, understood, and responded to upon receipt [24]; and they are relatively cheap, with high reachability because mobile phones are ubiquitous. We anticipated that SMS text messaging would allow simple, low-commitment participation in the survey. We considered the intrusiveness (or push factor) to be an advantage; however, one disadvantage is that messages are limited to 160 characters, after which additional payments fare required for every 160 characters. Our request to participate had many more characters; therefore, we had to pay 3 times the ordinary amount for each message. Another disadvantage is that more and more commercial organizations have started using SMS text messages for customer satisfaction surveys. This can lead to an overload of text messages considered to be spam.

In 2017, Pariyo et al [25] suggested that mobile phone surveys have the potential to become a powerful data collection tool to address public health challenges, such as those arising from noncommunicable diseases, in low- and middle-income countries. Ethical considerations in global mobile phone-based surveys of noncommunicable diseases have also recently been discussed, and a need for a broad conceptual framework for the ethical, legal, and societal issues associated with mobile phone surveys for noncommunicable disease risk factors was identified [26].

### Response Rate

In addition to randomized selection of participants, response rate is often considered to be an important factor in obtaining representative data. Our response rate (2305/11,000, 21.0%) was similar to those of 2 Norwegian eHealth research studies—a large population-based randomized controlled trial on smoking cessation with SMS text messaging or emails [27] and a web-based cross-sectional survey on diabetes [28]. We are satisfied that we achieved a response rate from the general population that was similar to that in these 2 surveys, which both had highly selected participants.

It is common for researchers to use monetary incentives (such as gift certificates or lottery tickets) to increase participation [29-31]. Instead of monetary incentives, we had intended to develop personalized feedback, based on national guidelines, for respondents. Though part of this feature was developed, due to time, monetary, and personnel constraints, we were not able to create detailed personalized responses for all possible combinations of answers to the survey. Since we did not complete this, the invitees were not informed about or given any options for personalized feedback. Most importantly, we were not able to examine if this would be an incentive to respond to the survey.

There is, as far as we know, no commonly accepted minimal response rate. In the UK Biobank cohort [32], there were close to 10 million invitees and the response rate was 5.45%. A response rate of 60% has been used as the threshold of acceptability for population-based face-to-face or postal surveys; however, this response rate can also cover up response bias [33] if the characteristics of nonrespondents differ from those of the respondents. However, if respondents are perfectly representative of the source population, a low response rate is not a problem. [33]. Low participation, high dropout, or high loss to follow-up may be expected features of eHealth research and likely should not be looked upon as failures [34].

We assume that our response rate would have been higher if our university, a renowned entity, had been the sender. Instead, the Norwegian Centre for E-health Research, which was established in 2016 (a few years before the survey), and Healthcom both appeared as senders of the initial SMS. A systematic review [35] found that response rates for paper questionnaires were higher if they originated from universities rather than from other sources such as commercial organizations. We have previously found that more specific titles to otherwise identical questionnaires influenced the response rate of mailed surveys [36]; therefore, in addition, the name of the survey—*Health and Disease*—was too general.

Another Norwegian eHealth study [37], which examined recruitment to public health surveys with electronic forms on 2 different platforms, found that (1) sampling from the national health website (30 000 invitees) yielded a response rate of 40.8%, whereas sampling from the National Population Registry (36 000 invitees) yielded a response rate of 41.5% and (2) there were systematic and pronounced differences in the responses of the 2 samples. Skogen et al [37] concluded that limiting recruitment to users of *Helsenorge (Health Norway)* services resulted in further selection problems.

Our survey was conducted in 2019 (before the COVID-19 pandemic) in Norway, and approximately 1 in 5 respondents (431/2209, 19.5%) reported being obese. In fall 2020, after the first national lockdown, the Norwegian Institute of Public Health conducted a pilot study, collecting data on risk factors for the 4 main noncommunicable diseases and several other topics [38], which noted as a limitation, that most questions in the survey had not yet been validated, but that this would be done at a later stage. As we did, they randomly sampled from each of the 11 counties, but they invited twice as many from each county and used both email and SMS text messaging to contact the invitees. The response rate to this survey [38] was 38.1%, and 16% of respondents reported being obese, and 59% of the men and 47% of the women reported being overweight or obese. We do not have a good explanation why we found greater percentages—63.9% (539/844) of men and 50.3% (686/1365) of women reporting being either overweight or obese.

Similar to our findings, (1) the 3 previously described eHealth studies [27,28,38] found that more women than men responded, and (2) the 2020 national survey [37] found that Trøndelag county had the highest response rate. One reason may be that the people residing in Trøndelag have been invited 4 times to the Trøndelag Health Study [11], which collected health survey data from the same geographic population [39].

In our survey, 10.7% (240/2235) reported smoking daily. For 2019, Statistics Norway reported overall daily smoking to be 9% for both sexes [40]. The proportion of men who reported being daily snus use in our study (153/850, 18.0%) was a little lower than that found by Statistics Norway in 2019 (20% [40]), while the proportion of women found by Statistics Norway (7% [40]) was the same as that found in our study (96/1368, 7.0%).

In our survey, 32.3% (237/735) men reported drinking alcohol more than once a week; the corresponding figure for Statistics Norway survey was 34%. In our survey, 7% (51/724) of men reported having consumed more than 6 units on the same occasion weekly; the corresponding figure for the 2019 Statistics Norway survey was 5%. In our survey, 23% (254/1119) of women reported drinking alcohol more than once a week; the corresponding figure for the 2019 Statistics Norway survey was 28%. In our survey, less than 2% reported having consumed more than 6 units on the same occasion weekly; the corresponding figure for the 2019 Statistics Norway survey was 3%.

A recent review [41] found that pilot or feasibility studies are still poorly reported, and only 8.9% of the 90 studies led to subsequent main studies. This study will continue to be developed as part of a larger project [42].

### Strengths

For a nationally representative study population, participants were sampled randomly from each of the 11 counties. Major assets were that our main survey could be answered in 5 minutes and comprised already validated questions.

### Limitations

The main limitation of our study was the low response rate. Other limitations include the use of self-reported information and that only individuals who had a smartphone could participate; however, since smartphone ownership exceeds 80% in Norway [14], not being eligible due to not having a smartphone is a minor concern. This may be a concern in other countries.

### Implications for Future Research

Short smartphone surveys have the potential to be used to monitor trends annually to identify high-risk groups for the 4 main noncommunicable diseases. This knowledge can subsequently be used for better targeting of interventions and in policy making, to meet the United Nations Sustainable Development Goal to reduce premature mortality from noncommunicable diseases by 33% by 2030 [4].

We encourage the further study of short mobile phone-based surveys regarding the modifiable risk factors for the 4 main noncommunicable diseases in high-, low-, and middle-income countries. Our study included only Norwegians, of whom the majority had more than high school level of education. Future studies should develop surveys that are easily recognized as research, have a well-known sender, and can be distinguished from spam. We recommend examining if an offer to the invitees for personalized feedback in response to their answers about the modifiable risk factors will increase overall participation.

### Conclusions

We successfully developed and piloted a smartphone-based information communication technology solution for collecting data annually on the four modifiable risk factors for the 4 main noncommunicable disease from a random sample of the Norwegian population; 1 in 5 responded, thus our secondary objective to collect nationally representative data may not have been achieved.

## Appendix

Multimedia Appendix 1 SMS text message request to participate in main and second survey.

Multimedia Appendix 2 Additional information given to the invitees after accessing the survey.

## Abbreviations

NOK: Norwegian Kroner
