# Supplementary material for: A Smartphone-Based Information Communication Technology Solution for Primary Modifiable Risk Factors for Noncommunicable Diseases: Pilot and Feasibility Study in Norway
Source: JMIR Form Res. 2022 Feb 25;6(2):e33636. doi: 10.2196/33636 (PMC8917437; doi:10.2196/33636)
Supplement: Multimedia Appendix 1 [file formative_v6i2e33636_app1.docx]

**Multimedia Appendix 1. SMS text messages request to participate in main and second survey.**

Request to participate main survey

“*Hi – full name. Do you want to participate in a national survey about health and disease? Your name is selected at random from the National Population Registry. It is voluntary to participate. Press here to get to the survey. . Link* [*https://services.healthcom.no/Q/?surevy_id=28&OTP=1513681173859.657*](https://services.healthcom.no/Q/?surevy_id=28&OTP=1513681173859.657) *to survey.*

*Thank you very much for your contribution! With kind regards,* *Norwegian Centre for E-health Research*.”.

Reminder, request to participate main survey

*“Hi – full name. This is a reminder. Do you want to participate in a national survey about health and disease? Your name is selected at random from the National Population Registry. It is voluntary to participate. Press here to get to the survey. Link* [*https://services.healthcom.no/Q/?surevy_id=28&OTP=1513681173859.657*](https://services.healthcom.no/Q/?surevy_id=28&OTP=1513681173859.657) *to survey.*

*Thank you very much for your contribution! With kind regards, Norwegian Centre for E-health Research*.”.

Non-responders, request to participate second survey

*“Hi – full name. You were one of those who chose not to participate in the national Health and Disease survey. Were any of these nine statements crucial to your decision? Link* [*https://services.healthcom.no/Q/?surevy_id=28&OTP=1513681173859.657*](https://services.healthcom.no/Q/?surevy_id=28&OTP=1513681173859.657) *to survey.*

*Sincerely, Norwegian Centre for E-health Research.”*
