# Supplementary material for: A Smartphone-Based Information Communication Technology Solution for Primary Modifiable Risk Factors for Noncommunicable Diseases: Pilot and Feasibility Study in Norway
Source: JMIR Form Res. 2022 Feb 25;6(2):e33636. doi: 10.2196/33636 (PMC8917437; doi:10.2196/33636)
Supplement: Multimedia Appendix 2 [file formative_v6i2e33636_app2.docx]

**Multimedia Appendix 2. Additional information given to the invitees after accessing the survey**

*“Reducing premature mortality from cardiovascular disease, cancer, chronic respiratory diseases and diabetes is a national goal. The Norwegian Centre for E-health Research is conducting this survey to contribute to this. It takes approx. 5 minutes to answer. It is voluntary to participate. All answers are stored and processed in accordance with the laws and regulations. Want to know more about the survey****?*** *Link to website; health and disease.no Thank you for your contribution! Norwegian Centre for E-health Research”.*
